# Factors predicting the intention of drug abuse avoidance among adolescents in Pinlaung Township, Myanmar: predictive correlational design

**DOI:** 10.1186/s12889-023-17419-4

**Published:** 2024-01-02

**Authors:** Nang Nwe Nwe Latt, Wimolnun Putdivarnichapong, Supapak Phetrasuwan, Nopporn Vongsirimas

**Affiliations:** 1 Student of Master of Nursing Science Program (International Program), Faculty of Nursing, Mahidol University, Bangkok, Thailand; 2 Department of Psychiatric and Mental Health Nursing, Faculty of Nursing, Mahidol University, Bangkok, 10700 Thailand

**Keywords:** Intention of drug abuse avoidance, Myanmar adolescents, Predictive factors

## Abstract

**Background:**

Adolescence is a critical transition period and is at high risk for drug/substance abuse. In Myanmar, drug use is common among adolescents and is a public health concern. There are no studies of drug abuse prevention among Myanmar adolescents. Intentions to avoid drug abuse can be a protective factor for preventing drug abuse among adolescents. This study investigated the effects of sex, parental history of drug/alcohol abuse, self-efficacy, parental marital status, and family functioning on the intention of drug abuse avoidance among Myanmar adolescents.

**Methods:**

This is a predictive correlational study. The Biopsychosocial model was used as the theoretical framework of this study. A convenient sampling method was used to collect data from 157 students aged 13–18 years in a government school, middle school level and high school level, Pinlaung Town, Southern Shan State, Myanmar during the COVID-19 pandemic and political protests. G* power software was used to calculate the sample size. Data was collected by four self-administered questionnaires: a socio-demographic questionnaire, Thai Family Functioning Scale (TFFS), General Self-Efficacy Scale (GSE), and Intention of Drug Avoidance Scale (IDAS). Multiple linear regression was employed to analyze the data.

**Results:**

Five predictors, including biological sex, parental history without drug/alcohol abuse, self-efficacy, parental marital status, and family functioning, explained 24.4% of the variance in the intention of drug abuse avoidance among Myanmar adolescents (*R* ^2^ = .244, F (5,151) = 9.738, *p* = .000). In addition, only three factors, family functioning (β = .31, *p* < .001), biological sex (β = -.25, *p* < .01), and self-efficacy (β = .16, *p* < .05) statistically and significantly predicted the intention of drug abuse avoidance among Myanmar adolescents.

**Conclusions:**

Family functioning, female gender, and self-efficacy predicted the intention of drug abuse avoidance among Myanmar adolescents in Pinlaung Township, Southern Shan State, Myanmar.

**Implications of this study:**

The results of this study have implications for all stakeholders through research, education, practice, and policymaking leading to improve the intentions of drug abuse avoidance among Myanmar adolescents. Furthermore, the results of this study specifically contribute to create psychoeducational intervention programs for increasing intention to avoid substance use by promoting family functioning and self-efficacy of adolescents. This is especially proper for male adolescents who have less intention to avoid substance use.

## Background

Nowadays, drugs have adverse effects on youth all over the world. Drug misuse, drug abuse, and the continuous use of drugs to produce pleasure, reduce stress, and change or avoid reality are inappropriate or unsafe use of drugs [1]. The initiation of drug use among adolescents has primarily arisen in 12–17-year-olds, with peak levels of drug use among those 18–25 years old [2]. More than 20 million adolescents age between 12 and 17 years old in the United States suffer from substance use disorders (SUDs) [3]. One multi-countries and cross-sectional study conducted in Cambodia, Indonesia, Laos, Malaysia, Myanmar, Philippines, Singapore, Thailand, and Vietnam found that adolescents in low or middle-income countries had a higher risk of frequent and irregular illicit drug use [4]. In ASEAN countries, admission to drug abuse treatment increased by 24.8% from 65.6 in 2019 to 81.9 per 100,000 population in 2020. The admission rate of drug abuse treatment increased in Myanmar from 10 per 100,000 in 2014 to 39.1 in 2019 and slightly decreased to 20 in 2020 [5].

Myanmar is facing drug challenges and concerns about adolescent addiction rates [6]. In 2019, 8,377 drug users were in treatment, and 25% of the users were under 25 years old [5, 7]. The Myanmar Narcotic Control Annual Report (2020) stated that among new cases, 0.02% of adolescents aged 10–14 years old and 2.81% of adolescents aged 15–19 years old were admitted for treatment in 2020. Additionally, in 2020, 446 youths under 18 years of age were arrested and prosecuted [8]. The increased arrest and treatment rates indicate the broad drug availability and higher rates of problematic drug use leading to adverse problems in Myanmar [9].

Adolescence is a period of transition allied with many physical and psychological challenges. Adolescents desire more freedom and independence in their daily lives [10]. Additionally, oversensitivity to the reward system, habits, and stress [11] leads to increased susceptibility to drug use and are at high risk of drug addiction and mental illness [12]. Adolescents are more likely to engage in unplanned or unintentional risky behaviors that may be reduced or prevented with deliberate preventative behaviors [13]. Early drug abuse has negative consequences that persist into adulthood [3]. Susceptibility to substance use depends on the critical combination of risk factors and the absence of important protective factors. Changing the balance between risk and protective factors of an individual and his/her surrounding environment is an important goal in prevention, therefore, protective factors have to be outweighed and greater than risk factors [14]. For the substance use prevention among adolescents, protective factors help to alleviate the effect of risk factors and likely to reduce the possibility of substance abuse [15]. Risk factors for drug abuse include gender (male) [16], comorbidities, exposure to significant negative growth [17], parental substance use [18–20], low parental education level, peer substance use [17], and single-parent families [16]. Some risk factors may be resistant or difficult to be changed. Therefore, focusing on and strengthening protective factors will be beneficial in managing and deterring drug misuse among at-risk and non-at-risk groups [21]. Protective factors promote drug use prevention, such as family functioning [14], living with both parents [16], intention to avoid drug abuse, high levels of fathers' awareness of drug abuse, individual traits of optimism [17], absence of parental substance abuse [22], and gender (female) [23]. Intentions to avoid drug abuse, including behavioral intentions would act as a protective factor for preventing drug abuse among adolescents [17]. Based on concept analysis, the intention of drug avoidance refers to self-control, motivation, commitment to living a drug-free life, having a negative attitude towards drugs and drug users, and have health literacy about drugs [24, 25]. Individuals with such intentions can say "No," take further steps to reach their goals, and be able to manage stress and risk situations [24]. There are several factors related to the intention to avoid drugs, including sex [16, 26, 27], parental history without substance abuse [22, 28, 29], self-efficacy [13, 30–35], parental marital status [36–38], and family functioning [14, 39–41].

The biopsychosocial model helps to assess biological, psychological, and social influences on overall health and health behavior [42]. George Libman Engel developed this model in 1977 [43]. It is reliable and used in medicine, including psychiatry, and drug abuse experts from a variety of health fields have clearly embraced this model [44, 45]. Skewes and Gonzalez (2013) proposed a biopsychosocial model of addictive behavior based on Engel's biopsychosocial model, in which biological factors such as genetic predisposition; psychological and cognitive factors such as outcome expectations, self-efficacy, and readiness for change; and social factors such as influences of family, peer, and intimate partner on substance abuse [45]. Most risk and protective factors for substance use can be classified into three main factors: biological factors, psychological factors, and social factors. Gurung (2014) also revealed that applying the biopsychosocial approach helps to understand the factors determining the health behavior of an individual [42]. Skewes and Gonzalez (2013) stated that a single factor cannot explain why some people do not progress into drug addiction even though some are facing substance abuse and dependence. Additionally, available evidence indicates that further studies are needed to uncover multiple factors that prevent substance use in adolescents [45]. However, it is clear that interactions of biopsychosocial factors which can be either protective or risk factors contribute to drug abuse prevention or drugs abuse depending on the direction of such factors.

**Fig. 1:**
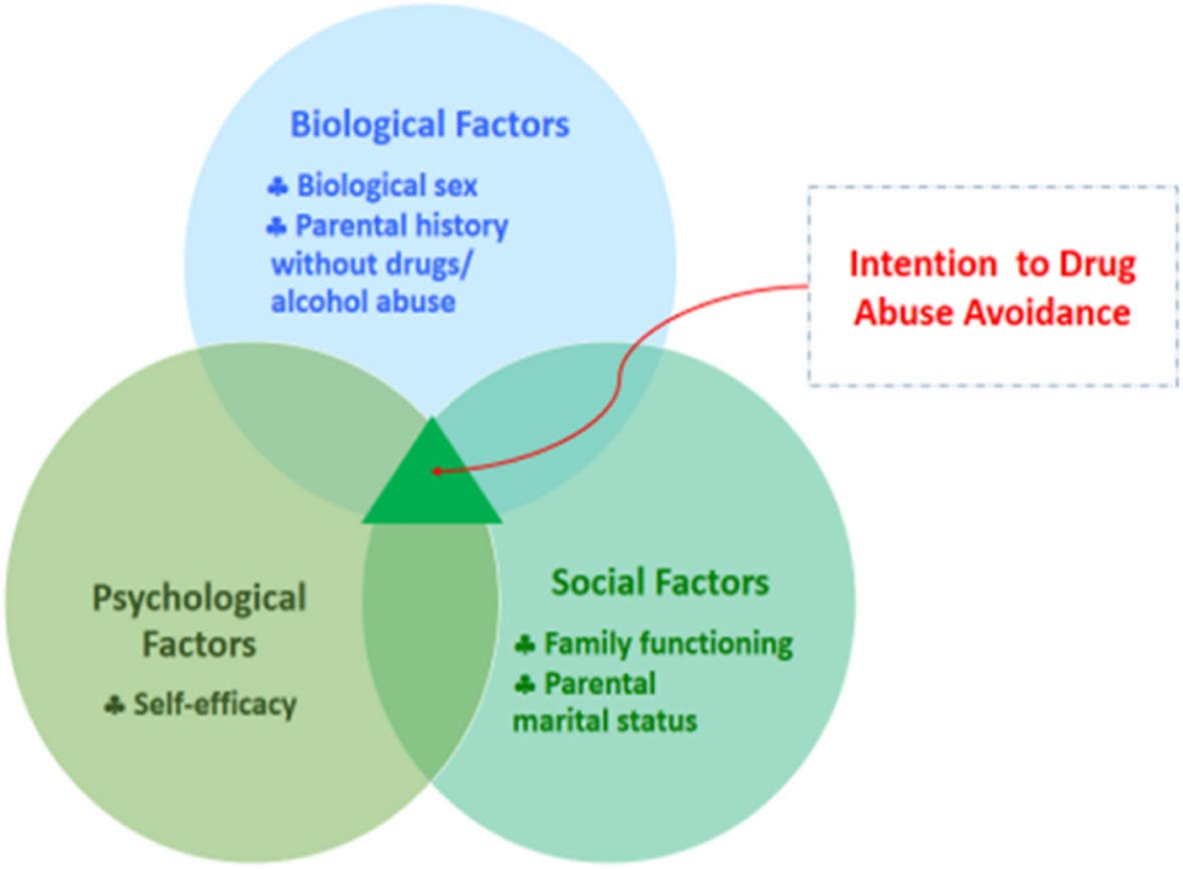
Biopsychosocial model of intention to drug abuse avoidance based upon a biopsychosocial model proposed by George L. Engel and Jon Romano (1977)

This study employs the biopsychosocial addiction model as the theoretical framework, as shown in Fig. 1. This model was constructed based upon a biopsychosocial model proposed by George L. Engel and Jon Romano (1977) [46]. The biopsychosocial model of addiction considered sex and genetic predisposition as *biological factors*. Biological sex, especially female gender, is a protective factor against substance use. This is because females have higher intentions to quit smoking than males [23] and have higher self-efficacy to avoid substance use [18]. As for genetic predisposition, Hicks and colleagues (2013) stated that the genetic inheritance of a spectrum of externalizing disorders is a common cause of similarity between parents and children in substance use disorders and antisocial behaviors [19, 20]. A drug-free parental history is considered to be the absence of a genetic predisposition to drug use in the family. This is because research shows that parental substance use is associated with adolescent SUD [20, 47]. Moreover, Altay and colleagues (2014) stated parental non-drug use as a protective factor against smoking and drinking [22]. Additionally, a study by Uzun and Kelleci (2018) found that adolescents with drug-abusing parents had low self-efficacy to avoid drug abuse [18].

For *psychological factors*, self-efficacy is a primary factor in this group. Self-efficacy is protective against substance use and other problem behaviors [13, 30, 32, 48]. It refers to an individual's perception of his/her confidence in achieving a particular behavior [49] and belief in his/her ability to manage difficult life experiences without the use of substances [50, 51]. Self-efficacy also affects physical and psychosocial functioning and education [48]. Numerous studies have revealed that perceived self-efficacy is a statistically significant predictor of intentions to avoid drug abuse. It can help to reduce drug use and resist peer pressure to use drugs [13, 31, 35]. As for social factors, such as parental marital status of adolescents, especially intact families, may play an important role in preventing drug use [16, 37]. Pisarska and colleagues (2016) found that the risk of smoking and drinking in adolescents decreased when living with both parents [16]. One study also revealed that intention to engage in risky behavior and rates of substance use among adolescents in intact families were lower than in single-parent families [37].

Another social factor is family functioning which has a significant impact on the prevention of substance use [39, 40]. Family functioning is well defined as the set of key family system processes related to evaluation, function, and performance [52]. Warm and supportive parenting strategies and positive family functioning contribute to achieve healthy social, emotional, and cognitive development among adolescents [53] and family satisfaction [54]. Moreover, pro-social family involvement is a protective factor for adolescents [41]. Fang and colleagues (2010) reported that self-efficacy, refusal skills, and family rules against drug use have been improved after providing a family-focused, web-based substance abuse prevention program to Asian American teenage girls. They also indicated lower intentions to use substances in the future [40].

Additional evidence from a systematic literature review indicates that adolescent smoking was reduced by intensive family-based interventions that focus on family functioning [39]. This successful intervention is consistent with Kao et al.'s study, which revealed that if adolescents are highly satisfied with their family functioning, risky behavior will be reduced [55]. Shek and Leung also stated that adolescents from families with higher levels of positive life satisfaction were less likely to engage in risky behaviors and problem behaviors [56]. Therefore, as a social factor, healthy family functioning may act as a protective factor in increasing intentions to avoid substance use and preventing adolescents from substance use and misuse.

Understanding the biological, psychological, and social factors that protect against drug and alcohol use, specifically, factors that can predict intentions to avoid drug use are of great importance to all stakeholders involved in substance use prevention. However, such factors studied in Western and North American countries may differ from those in Asian countries due to cultural differences, economic and social context, and government policy and support. Myanmar is considered a collectivistic culture, unlike Western and North American countries which are believed to be individualistic culture [57]. Family members with collectivistic cultures tend to focus on interconnectedness among family members, the needs, and goals of the family as a whole. [58, 59]. Furthermore, Buddhism has become dominant in Myanmar. Most of Myanmar families use Buddhist teachings to guide their lives and behaviors especially five Precepts of Buddhist teachings and of these five Precepts urges persons to avert from alcohol drinking and substance abuse [60]. Buddhism has much influence on the development of Myanmar cultures. Especially for women, the use of drugs or recreational substances that do not cause problems is prohibited in rural and urban communities in Myanmar. Myanmar society views this behavior as inappropriate for women [61].

Until now, there is a paucity of evidence on drug use prevention and factors predicting intentions to avoid drug misuse among adolescents in Myanmar. Therefore, this study aimed to study whether biological sex, parental history without drugs/alcohol abuse, self-efficacy, marital status of parents, and family functioning can predict the intention of drug abuse avoidance among adolescents in Pinluang Township, Myanmar. The research hypothesis was biological sex, parental history without drugs/alcohol abuse, self-efficacy, parental marital status, and family functioning could predict the intention of drug abuse avoidance among adolescents in Pinlaung Township, Myanmar.

The results of this study will contribute to deepen our understanding of the factors that predict intentions of drug abuse avoidance among adolescents in Myanmar. It may lead to the development of preventive intervention programs to avoid drug use. The results may also influence different sectors of society, including health professionals, educators, social workers and staffs in drug prevention agencies. Furthermore, it will be helpful for public health policymakers to engage with all stakeholders, including youth, families, school personnel, communities, and other organizations such as the Central Committee for Narcotics Control (CCDAC) and the Anti-Drug Association of Myanmar (MANA) in youth protection from substance use through working together to create a safe environment from substance use for adolescents.

## Methods

###  Study design

This study was a predictive, cross-sectional, and correlational study that used convenient sampling to examine the prediction of the intention of drug abuse avoidance by biological sex, parental history without drugs/alcohol abuse, self-efficacy, parental marital status, and family functioning among adolescents in Pinlaung Township, Myanmar. The data was collected from January to March 2022.

###  Sample and procedure

The data was collected from one public school which has 2 levels of study, middle school students and high school students, aged between 13- 18 in Pinlaung Township, Southern Shan State, Myanmar. Adolescents diagnosed with anxiety, depression, bipolar disorder, schizoaffective disorder, schizophrenia, reading disabilities, and learning disabilities (LD) were excluded from the study.

The school offers education from grade 1 to grade 11 and accepts both male and female students. Initially, the researcher intended to collect data from 25 students in each grade. In fact, data collection depended on the student's address being accessible to the researcher. Therefore, more than 25 students from each of the 8th, 10th, and 11th grades participated in the study. The detail was described in Table 1.

**Table 1:** Total number of students in each grade participated in the study

Student Grade	Total number of students (Academic year 2020–2021)			Total number of students participated in the study
	**Male**	**Female**	**Total**	
Grade 7	107	157	264	25
Grade 8	53	57	110	38
Grade 9	41	56	97	25
Grade 10	70	87	157	33
Grade 11	73	137	210	36
**Total**	344	494	838	157

G* power software was used to calculate the sample size. The effect size was calculated using a correlation coefficient of 0.28 obtained from the previous study of Li and Wang (2006). It revealed a strong correlation between self-efficacy avoidance of environmental tobacco smoke (ETS) and behaviors to avoid ETS among adolescents in southern Taiwan [62]. The variables and a population of this study were similar to those of the present study. The result of using G* power software to calculate sample size with a value for probability error (α) of 0.05, power (1-β) as 0.80, effect size as 0.08, five independent variables, and multiple linear regression was 157 participants.

### Instruments

The researchers developed the Socio-Demographics Questionnaire to collect information about the demographic characteristics of each adolescent. Other instruments, including the General Self-Efficacy (GSE) Scale) [63], the Thai Family Functioning Scale (TFFS) [64], and the Intention to Drugs Avoidance Scale (IDAS) [25] were used to collect data. The General Self- Efficacy (GSE) scale was used to measure the self-efficacy among Myanmar adolescents. The German version of the GSE scale was initially created by Matthias Jerusalem and Ralf Schwarzer in 1979. It consisted of 20 items to assess a general sense of perceived self-efficacy of the general adult population, including adolescents. It was later revised to 10 items and adapted to 32 other languages [63]. The GSE scale is a self-report measure of self-efficacy, with a four-point Likert scale for responses. The total score is calculated by combining scores of all items and ranges between 10 and 40, with a higher score indicating more self-efficacy [65]. The cutoff point is 20, therefore, scores greater than 20 indicate high levels of general self-efficacy while scores below 20 indicate low self-efficacy [66].

Thai Family Functioning Scale (TFFS) was developed by Wannapa Phetecharapan Suttiamnuaykul (2002) [64]. The TFFS was developed based upon Thai culture and socioeconomic environment. There are 30 items: 12 items of cohesion, 8 items of communication/ feeling expression, and 10 items of problem solving. The total score ranges between 0 and 90. Interpretation of family functioning is classified as a score of 0 – 30 indicating unhealthy or poor family functioning, a score of 31 – 60 indicating moderate family functioning, and a score of 61 – 90 indicating healthy or good family functioning. This study interpreted a total score above 30 as healthy family functioning and a score of 30 or less than 30 as unhealthy family functioning.

The Intention to Drugs Abuse Avoidance Scale (IDAS) was used to measure the intention to drugs abuse avoidance among Myanmar adolescents in this study. It was developed by Suwanchinda, Suttharangsee, and Kongsuwan (2019) [25]. Adolescents rated their answers on a 5-point Likert scale, with 1 meaning not at all true and 5 meaning extremely true. The IDAS consists of 22 items divided into 2 factors: 16 items of desire and intention to avoid drugs and 7 items of readiness to avoid drugs. The levels of interpretation of intentions to avoid drug use are as follows: highest intention to avoid drugs (scores 4.21–5.00 points), high intention to avoid drug abuse (scores 3.41–4.20 points), moderate intention to avoid drug use (score 2.61–3.40), low intention to avoid drug abuse (score 1.81–2.60), and least intention to avoid drug abuse (score 1.00–1.80) respectively.

The researcher requested permission from all copyrights of the instruments to use and translate the instruments. Back-and-forth translation process was used to ensure the validity of the translation. These instruments have trustworthy validity and reliability as reported in Table 2. Additionally, all instruments were tested for content validity by three Myanmar mental health and psychiatric experts to determine all aspects of the items, constructs, and behaviors they were designed to measure as well as the appropriateness of its use in Myanmar culture and society. The reliability of the instrument was tested by collecting data from 30 students with similar characteristics to the sample in this study.

**Table 2:** Psychometric properties of the employed instruments

Instruments	Types of Validity		Types of Reliability	
	**Studied by the developer(s) of the instrument**	**Studied by the present study**	**Studied by the developer(s) of the instrument**	**Studied by the present study (** ***n*** ** = 30)**
General Self-Efficacy (GSE) scale (10-item version) [63]	- Concurrent validity - Prognostic validity	- Content validity	- Internal consistency, samples from 23 nations, Cronbach's alphas ranged from .76 to .90, with the majority in the high .80 s	- Internal consistency, Cronbach's alpha = 0.725
Thai Family Functioning Scale (TFFS) [64]	- Content validity - Construct validity - Discriminant validity - Predictive validity of concurrent events	- Content validity	- Internal consistency, Cronbach's alpha = 0.88 - Test–retest reliability over one week = .80	- Internal consistency, Cronbach's alpha = 0.863
Intention to Drugs Avoidance Scale (IDAS) [25]	- Content validity - Construct validity	- Content validity	- Internal consistency, Cronbach's alpha = 0.94 - Test–retest reliability over two weeks, *r* = .77 (*p* = < .01)	- Internal consistency, Cronbach's alpha = 0.859

### Data collection

After receiving approval from the Institutional Review Board (IRB), Faculty of Nursing, Mahidol University and received permission from the relevant public secondary school to collect data, the researcher met with students and parents at their homes to explain details of the study. Schools have been closed due to the COVID-19 pandemic [67] and protests that have started since 2021 against the coup in Myanmar and has been known locally as the Spring Revolution [68]. If they agreed to participate in this study, the adolescent and parent/guardian voluntarily signed the informed consent form and later the participants completed the questionnaires. This data collection process was performed following the ethical standards of the Institutional Review Board.

### Data analysis

The data were analyzed using the Statistical Package for the Social Science (SPSS) software version 26. Descriptive statistics were used to report participant characteristics and study variables. Multiple linear regression was used to examine the predictive power of independent variables on levels of intention to avoid drug abuse among adolescents. The assumptions of multiple linear regression were tested before the data analysis was performed.

## Results

The findings of demographic data indicated that 54.1% were female and 45.9% were male. Most families were middle-income families. Adolescents reported that 31.8% of their parents used substances, mostly tobacco (*n* = 31, 19.8%). In addition, 23.6% of adolescents also had a history of drug use. Furthermore, most adolescents reported that they lived with both parents (*n* = 109, 69.4%) and their parents lived together (*n* = 123, 78.3%). Details are shown in Table 3**.**

**Table 3:** Socio-demographic characteristics of the Myanmar adolescents (*N* = 157)

Socio-demographic Characteristics	n	%
**Sex**		
Male	72	45.9
Female	85	54.1
**Age (year)**		
13	45	28.7
14	27	17.2
15	38	24.2
16	32	20.4
17	9	5.7
18	6	3.8
**Education**		
Grade 7	25	15.9
Grade 8	38	24.2
Grade 9	25	15.9
Grade 10	33	21
Grade 11	36	22.9
**Religious**		
Buddhism	154	98.1
Islam	3	1.9
**Father educational level**		
College	19	12.1
High School	61	38.9
Middle School	42	26.8
Primary School	26	16.6
Other	9	5.7
**Mother educational level**		
College	28	17.8
High School	49	31.2
Middle School	37	23.6
Primary School	32	20.4
Other	11	7
**Family income per month (Kyats is Myanmar currency.)**		
< 100,000 kyats (< $47.60)	33	21
100,000—200,000 Kyats ($47.60—$95.21)	49	31
200,000—300,000 Kyats ($95.21 -$142.81)	48	30.6
> 300,000 Kyats (> $142.81)	27	17.2
**Parental substance use**		
Yes	50	31.8
No	107	68.2
**Types of substances used by parents**		
Tobacco	31	19.8
Alcohol	25	16
Opioids	1	.6
**Student history of non-medical abuse**		
Yes	37	23.6
No	120	76.4
**Students are living with**		
Both parents	109	69.4
Mother only	18	11.5
Father only	7	4.5
Relative	11	7
Other	12	7.6
**Parental marital status**		
Living together	123	78.3
Separated	8	5.1
Divorced	15	9.6
Deceased Father	11	7

 Most adolescents had healthy or good family functioning with a mean of 64.64 (SD = 10.28) and high self-efficacy with a mean score of 29.75 (SD = 4.49), as shown in Table 4.

**Table 4:** Descriptive statistics of the intention of drugs abuse avoidance, self-efficacy, and family functioning (*N* = 157)

Variables	Possible range	Actual range	Mean	SD	Interpretation
**Intention of Drug Abuse Avoidance** (22 items)	1–5	2.36–5	4.32	.53	The most intention to avoid drugs abuse
**Self-efficacy** (10 items)	10–40	20–40	29.75	4.49	High level of general self-efficacy
**Family Functioning** (30 items)	0–90	33–87	64.64	10.28	Healthy or good family functioning

As presented in Table 5, biological sex had a significant negative correlation with the intention of drug abuse avoidance among adolescents (*r* = -0.30, *p* < 0.001). Meanwhile, there was a significant positive correlation between the intention of drug abuse avoidance and family functioning and self-efficacy (*r* = 0.38, *p* < 0.001 and *r* = 0.24, *p* < 0.001, respectively).

**Table 5:** Relationship between variables and intention of drug abuse avoidance among adolescents

	**Variables**	**Correlation coefficient (r) between the intention of drugs abuse avoidance and variable**
1	Biological sex	-.30^a^
2	Parental history without drugs/alcohol abuse	-.02
3	Self-efficacy	.24^a^
4	Parental marital status	.04
5	Family functioning	.38^a^

All correlation coefficients are Spearman Rank correlations

^a^Correlation is significant at the 0.01 level (2-tailed)

Multiple regression analysis shown in Table 6 revealed that all five variables explained 24.4 percent of the variance in intention of drug abuse avoidance (*R* ^2^ = 0.244, F (5, 151) = 9.738, *p* = 0.000). However, there were only 3 variables: family functioning (β = 0.31, *p* < 0.001), biological sex (β = -0.25, *p* < 0.01), and self-efficacy (β = 0.16, *p* < 0.05) statistically significantly predicted the intention of drug abuse avoidance. Meanwhile, parental history without drugs/alcohol abuse (β = 0.08, *p* = 0.264.) and parental marital status (β = -0.03, *p* = 0.700) did not significantly predict the intention of drug abuse avoidance among adolescents. Furthermore, the F-ratio indicated that the overall regression model of this study was a good fit for the data. The regression model suitable for this study is: Intention of Drugs Abuse Avoidance = 2.85- 0.26 x (sex) + 0.02 x (self-efficacy) + 0.02 x (family functioning).

**Table 6:** Multiple regression analysis (enter method) of variables and intention of drug abuse avoidance among adolescents

Variables	B	SE	β	t	Sig
Constant	2.85	.32		9.01	.000
Biological sex	-.26	.08	-.25	-3.30	.001
Parental history without drugs/alcohol abuse	.09	.08	.08	1.12	.264
Self-efficacy	.02	.01	.16	2.02	.045
Parental marital status	-.04	.09	-.03	-.39	.700
Family functioning	.02	.00	.31	3.80	.000
	*R* = .494	*R* ^2^ = .244	Adjusted *R* ^2^ = .219	F(5,151) = 9.738	*p* = .000

## Discussion

For this study, biological sex is a statistically significant predictor of the intentions to avoid drug abuse in adolescence. The results showed that females were more committed than males to avoid drug abuse, which is consistent with the results of previous studies [18, 23, 26, 27]. This is due to the awareness of the physical harms and social rejection of Thai women taking drugs [23] and the choice to avoid taking drugs including parental prohibitions among Danish youth [27] leading to lower intentions to use drugs and greater prevention of drug abuse. In this study Myanmar female teenagers are highly motivated to avoid drug abuse because Myanmar women are expected to be virtuous. Both urban and rural Myanmar communities view the use of drugs or non-problematic substances as unacceptable behavior for women [61]. In addition, one of the five moral precepts of Buddhism which is the main religion in Myanmar advises lay people to abstain from the use of intoxicating substances [69].

However, the result of this study showed that a parental history of no drug/alcohol abuse was not statistically significant predictor of the intentions to avoid drug abuse among Myanmar adolescents. This finding was consistent with the results of the previous study on Chinese youth's intention to quit smoking [29]. The reason for this may be that strong family cohesion can reduce the risk of drug use among teenagers [70]. For this study, the majority of parents lived together and the majority of teenagers lived with their parents and had healthy family functioning, all of which created family cohesion. This may also be explained by the fact from the demographics of the sample that the substances most commonly abused by parents are tobacco and alcohol which are not considered as serious as illegal substances and are accepted as a way of life in Myanmar culture. In addition, in Myanmar society adolescents are always advised to avoid drug use by those who involved with them, such as parents, other family members, teachers, government officials, and international non-profit organizations. This is similar to Hong Kong where parents and teachers forbid teenagers from taking drugs and smoking [29]. It can be noted that these two societies are in Asia which is considered as collectivist cultures. The result of this study is in contrast to the result of a study that found that parental alcohol dependence and parental drug use were significantly associated with later substance use among adolescents in the United States [70] which is viewed as individualistic cultures. However, alcohol abuse is less severe than alcohol dependence.

The result of this study indicated that self-efficacy as a psychological factor was a statistically significant predictor of the intention of drug abuse avoidance among Myanmar adolescents. This predictive result is consistent with another study which revealed that higher self-efficacy significantly predicted lower intentions of cannabis use and lower intentions to start consuming cannabis in the future [33]. Similarly, other studies also revealed that self-efficacy may encourage adolescents to plan for avoiding drug abuse [17, 30, 31, 49]. The supporting reason for this study may be that most of the adolescents in this study had high self-efficacy. Therefore, daily stressful situations can be resolved [71] in order to reduce habits that are harmful to health and create positive health habits leading to a lower likelihood of drug use [72]. Additionally, self-efficacy empowers personal resources [73], increases optimistic beliefs [65], and influences the impact of substance use among peers [74].

Regarding social factors, the results of this study show that parental marital status did not predict the intentions to avoid drug abuse among Myanmar adolescents. This result is inconsistent with the results of a previous study [37] which indicated that intact families act as a protective factor against substance use in adolescents. Another study also reported that adolescents facing recent parental divorce encountered stressful life conditions, higher levels of depression and anxiety, and increasing alcohol consumption [36]. However, this study investigated behaviors related to drinking and smoking, which is not a study of intentions to avoid drug/alcohol abuse. In fact, adolescents may already have drinking and smoking habits and when family problems arise, they may drink and/or smoke more and more. In addition, these studies were conducted in individualistic cultures which is in contrast to the collectivistic culture of Myanmar. Collectivism emphasizes the significance of societal groups, while individualism concentrates on the rights and business of each person [75]. Myanmar society is considered as collectivist culture which stresses interconnectedness among family members and the willingness to fulfill the needs and desires of each family member [59]. Therefore, Myanmar children and adolescents, whether from intact or non-intact families, receive care, love, and supervision from their parents and other family members causing them to have proper behaviors, including avoiding substance abuse.

Another result regarding social factors is family functioning, which statistically significantly predicted the intention of drug abuse avoidance among adolescents. This was consistent with the results of many studies [40, 55, 76]. It could be explained that healthy families with biological parents encourage positive youth development outcomes and reduce intention to engage in risky behaviors [56]. Additionally, healthy family functioning may improve adolescent's self-image leading to reduce risk of substance use [77], reduce substance use as a coping mechanism, improve self-esteem, and reduce depressive symptoms [78].

## Limitations

A limitation of this study may be related to the fact that drug use is not accepted by society. Thus, adolescents may report intentions to avoid substance use in an effort to present themselves in a socially acceptable way. Additionally, this study assessed family functioning only from the perspective of adolescents. Furthermore, this study used a convenient sampling method and collected data from only one government school due to the Covid-19 pandemic [67] and the protests in Myanmar. It was difficult to collect data during this challenging time. Myanmar government has used several countermeasures, including internet and media blackouts, the arrest and criminal prosecution of the protesters, the spread of disinformation, the deployment of pro-military protesters and instigators, and the violent use of force to suppress protests [68]. All of these situations have caused people to stop doing their normal daily activities. The schools were closed. People have been afraid to go out and talk to strangers. An additional limitation is that adolescents who dropped out of school or worked or studied in private schools were not investigated. Therefore, these limitations may affect the generalizability of the results of this study to adolescents in other settings and in other geographic regions.

## Recommendations

The qualitative approach is recommended for future studies to obtain deeper exploration and information. In addition, future studies should assess the perception of the parents regarding family functioning as well as acquire more precise information. Furthermore, additional research should investigate the intention of drug abuse avoidance of adolescents who are dropouts from school and the students of private schools. Further studies will provide a deeper and broader understanding of drug abuse prevention and the results can be generalized to adolescents across the region and across the country. Additional studies should examine other factors that may also predict the intention of drug abuse avoidance among adolescents. Finally, mixed method approach is recommended for future studies in addition to qualitative approach.

## Conclusion

**Fig. 2:**
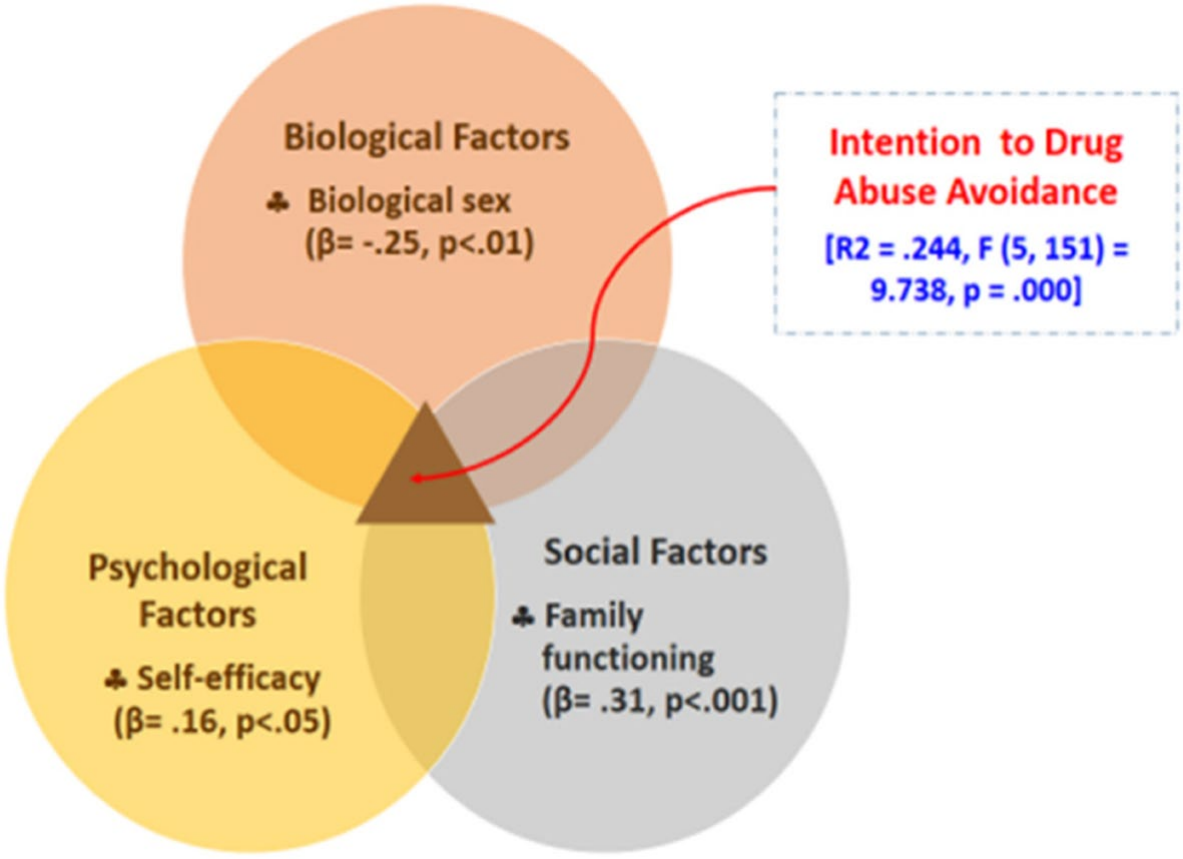
Biopsychosocial model of intention to drug abuse avoidance among adolescents in Pinlaung Township, Myanmar

Adolescence is a critical life transition period with the highest substance abuse tendency [2]. Adolescents also have poor judgment and a lack of impulse control. This is because various self-regulatory executive functions are still maturing, and the brain undergoes considerable development during adolescence [11]. This study investigated the factors that could predict the intention of drug abuse avoidance of middle and high school students in Pinlaung Township, Myanmar based upon the biopsychosocial model as theoretical framework of this study. The results showed that biological sex, parental history without drugs/alcohol abuse, self-efficacy, parental marital status, and family functioning could predict 24.4% of the intention of drug abuse avoidance. There are only three variables: family functioning, biological sex, and self-efficacy that can statistically significantly predict intentions of drug abuse avoidance, as shown in Fig. 2.

The findings of this study are beneficial for all stakeholders involving substance abuse prevention through research, education, practice, and policymaking by developing program to promote intention of drug abuse avoidance, issuing education and public health policies relating to substance abuse prevention, studying other predictive variables of intention of drug abuse avoidance, and conducting researches in other settings. The results of this study make a special contribution to the creation of psychoeducational intervention programs that promote family functioning and adolescent self-efficacy. This is especially true in men who have little intention to avoid drug abuse.

## Data Availability

The datasets used and/or analyzed during the current study available from the corresponding author on reasonable request.

## Abbreviation

UNODC: United Nations Office on Drugs and Crime
LD: Learning disability
ETS: Environmental Tobacco Smoke
GSES: General Self-Efficacy Scale
TFFS: Thai Family Functioning Scale
IDAS: Intention to Drugs Avoidance Scale
